# Author Correction: Mature Andean forests as globally important carbon sinks and future carbon refuges

**DOI:** 10.1038/s41467-021-23955-7

**Published:** 2021-06-08

**Authors:** Alvaro Duque, Miguel A. Peña, Francisco Cuesta, Sebastián González-Caro, Peter Kennedy, Oliver L. Phillips, Marco Calderón-Loor, Cecilia Blundo, Julieta Carilla, Leslie Cayola, William Farfán-Ríos, Alfredo Fuentes, Ricardo Grau, Jürgen Homeier, María I. Loza-Rivera, Yadvinder Malhi, Agustina Malizia, Lucio Malizia, Johanna A. Martínez-Villa, Jonathan A. Myers, Oriana Osinaga-Acosta, Manuel Peralvo, Esteban Pinto, Sassan Saatchi, Miles Silman, J. Sebastián Tello, Andrea Terán-Valdez, Kenneth J. Feeley

**Affiliations:** 1 Departamento de Ciencias Forestales, Universidad Nacional de Colombia Sede Medellín, Medellín, Colombia; 2 Grupo de Investigación en Biodiversidad, Medio Ambiente y Salud -BIOMAS - Universidad de Las Américas (UDLA), Quito, Ecuador; 3 Department of Plant and Microbial Biology, University of Minnesota, Saint Paul, MN USA; 4 School of Geography, University of Leeds, Leeds, UK; 5 Centre for Integrative Ecology, School of Life and Environmental Sciences, Deakin University, Melbourne, VIC Australia; 6 Instituto de Ecología Regional (IER), Universidad Nacional de Tucumán (UNT) - Consejo Nacional de Investigaciones Científicas y Técnicas (CONICET), Tucumán, Argentina; 7 Herbario Nacional de Bolivia (LPB), La Paz, Bolivia; 8 Missouri Botanical Garden, St. Louis, MO USA; 9 Center for Conservation and Sustainable Development, Missouri Botanical Garden, St. Louis, MO USA; 10 Living Earth Collaborative, Washington University in Saint Louis, St. Louis, MO USA; 11 Plant Ecology and Ecosystems Research, University of Gottingen, Gottingen, Germany; 12 Centre of Biodiversity and Sustainable Land Use (CBL), University of Gottingen, Gottingen, Germany; 13 Environmental Change Institute, School of Geography and the Environment, University of Oxford, Oxford, UK; 14 Facultad de Ciencias Agrarias, Universidad Nacional de Jujuy, Jujuy, Argentina; 15 Université du Quebec a Montreal, Montreal, QC Canada; 16 Department of Biology, Washington University in St. Louis, St. Louis, MO USA; 17 Consorcio para el Desarrollo Sostenible de la Ecorregión Andina (CONDESAN), Quito, Ecuador; 18 Columbus State University, University System of Georgia, Columbus, GA USA; 19 Carbon Cycle and Ecosystems, Jet Propulsion Laboratory, California Institute of Technology, Pasadena, CA USA; 20 Center for Energy, Environment and Sustainability, Winston-Salem, NC USA; 21 Centro Jambatú de Investigación y Conservación de Anfibios, Quito, Ecuador; 22 Biology Department, University of Miami, Coral Gables, FL USA

**Keywords:** Carbon cycle, Ecosystem ecology

Correction to: *Nature Communications* 10.1038/s41467-021-22459-8; Published online 09 April 2019.

The numbering in the reference list was incorrect in the original article through numbers 29 to 65. This has now been amended.

