# Supplementary material for: Mature Andean forests as globally important carbon sinks and future carbon refuges
Source: Nat Commun. 2021 Apr 9;12:2138. doi: 10.1038/s41467-021-22459-8 (PMC8035207; doi:10.1038/s41467-021-22459-8)
Supplement: Supplementary file 1 — Supplementary Information [file 41467_2021_22459_MOESM1_ESM.pdf]

Supplementary Information

Mature Andean forests as globally important carbon sinks and future carbon refuges

Alvaro Duque[1*], Miguel A. Peña[1], Francisco Cuesta[2], Sebastián González-Caro[1], Peter Kennedy[3], Oliver L. Phillips[4], Marco Calderón-Loor[2,5], Cecilia Blundo[6], Julieta Carilla[6], Leslie Cayola[7,8], William Farfán-Ríos[9,10], Alfredo Fuentes[7,8], Ricardo Grau[6], Jürgen Homeier[11,12], María I. Loza-Rivera[7,8,9], Yadvinder Malhi[13], Agustina Malizia[6], Lucio Malizia[14], Johanna A. Martínez-Villa[15], Jonathan A. Myers[16], Oriana Osinaga-Acosta[6], Manuel Peralvo[17], Esteban Pinto[2,18], Sassan Saatchi[19], Miles Silman[20], J. Sebastián Tello[9], Andrea Terán-Valdez[21], Kenneth J. Feeley[22]

Affiliations:

[1] Departamento de Ciencias Forestales, Universidad Nacional de Colombia Sede Medellín, Medellín, Colombia

[2] Grupo de Investigación en Biodiversidad, Medio Ambiente y Salud -BIOMAS - Universidad de Las Américas (UDLA) Quito, Ecuador

[3] Department of Plant and Microbial Biology, University of Minnesota, Saint Paul, MN USA

[4] School of Geography, University of Leeds, Leeds LS2 9JT, United Kingdom

[5] Centre for Integrative Ecology, School of Life and Environmental Sciences, Deakin University, Melbourne, Australia

[6] Instituto de Ecología Regional (IER), Universidad Nacional de Tucumán (UNT) - Consejo Nacional de Investigaciones Científicas y Técnicas (CONICET), Tucumán, Argentina

[7] Herbario Nacional de Bolivia (LPB), La Paz, Bolivia

[8] Missouri Botanical Garden, St. Louis, MO, USA

[9] Center for Conservation and Sustainable Development, Missouri Botanical Garden, St. Louis, MO, USA

[10] Living Earth Collaborative, Washington University in Saint Louis, St. Louis, MO 63130 USA

[11] Plant Ecology and Ecosystems Research, University of Gottingen, Gottingen, Germany

[12] Centre of Biodiversity and Sustainable Land Use (CBL), University of Gottingen, Gottingen, Germany

[13] Environmental Change Institute, School of Geography and the Environment, University of Oxford, South Parks Road, Oxford OX1 3QY, United Kingdom

[14] Facultad de Ciencias Agrarias, Universidad Nacional de Jujuy, Jujuy, Argentina

[15] Université du Quebec a Montreal, Montreal, Canada.

[16] Department of Biology, Washington University in St. Louis, MO, USA

[17] Consorcio para el Desarrollo Sostenible de la Ecorregión Andina (CONDESAN), Quito, Ecuador

[18] Columbus State University, University System of Georgia, USA.

[19] Carbon Cycle and Ecosystems, Jet Propulsion Laboratory, California Institute of Technology, Pasadena, CA, USA

[20] Center for Energy, Environment and Sustainability, Winston-Salem, NC, USA

[21] Centro Jambatú de Investigación y Conservación de Anfibios, Quito, Ecuador.

[22] Biology Department, University of Miami, Coral Gables, FL, USA

*Correspondence to: ajduque@unal.edu.co. Departamento de Ciencias Forestales, Universidad Nacional de Colombia Sede Medellín. Cra 65 #59A-109, Medellín. Colombia.

Supplementary Figures

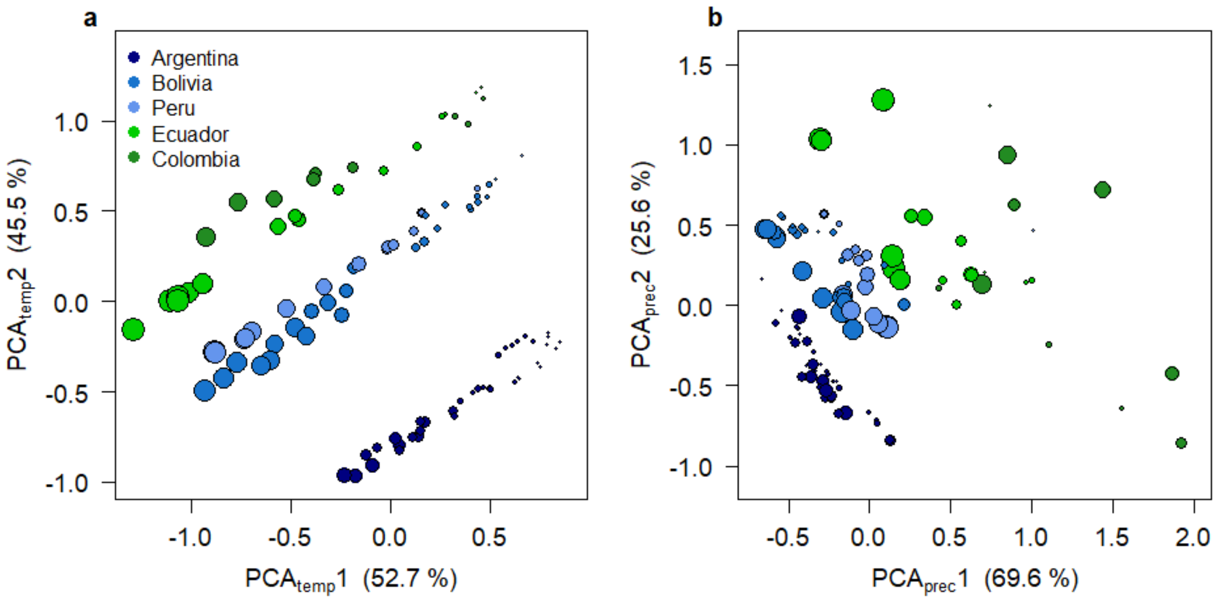

Supplementary Figure 1. Principal components analysis (PCA) of the climatic variables employed to define the gradient of climate variation across the subtropical and tropical Andes. Data were obtained from the CHELSA database[46]. Panel **a**: Temperature. Panel **b**: Precipitation. The size of the circle is proportional to elevation (m asl). Variable loadings are presented in the Supplementary Table 4.

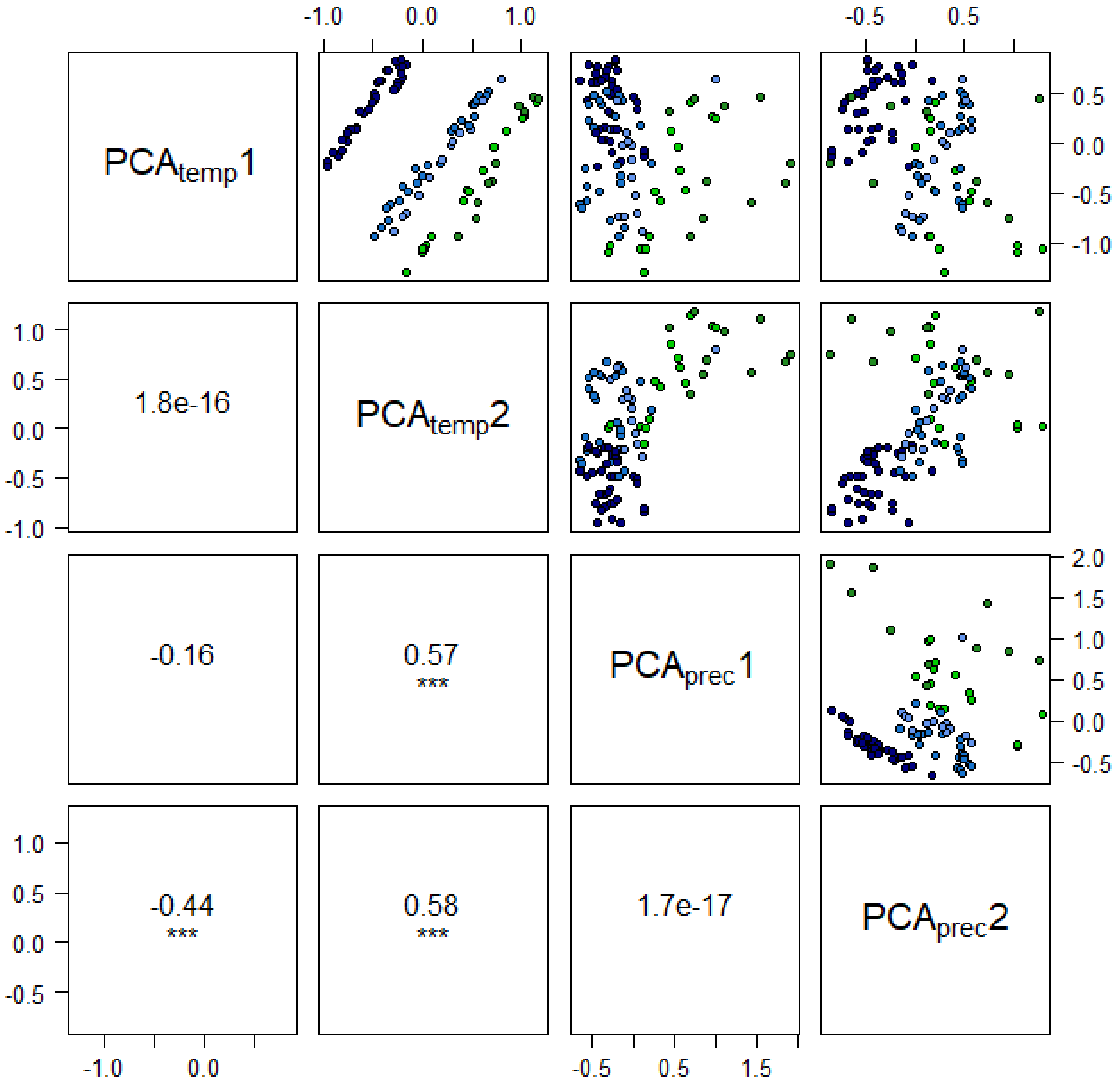

Supplementary Figure 2. Pearson correlation coefficients between the two first axes derived from the principal component analysis (PCA) applied to the data of temperature (11 variables) and the two first PCA axes applied to the precipitation data (8 variables) across the 119 plots established in the subtropical and tropical Andes. The associated probability of the correlations was defined as follows: *: $P \leq 0.05$. **: $P \leq 0.01$. ***: $P \leq 0.001$.

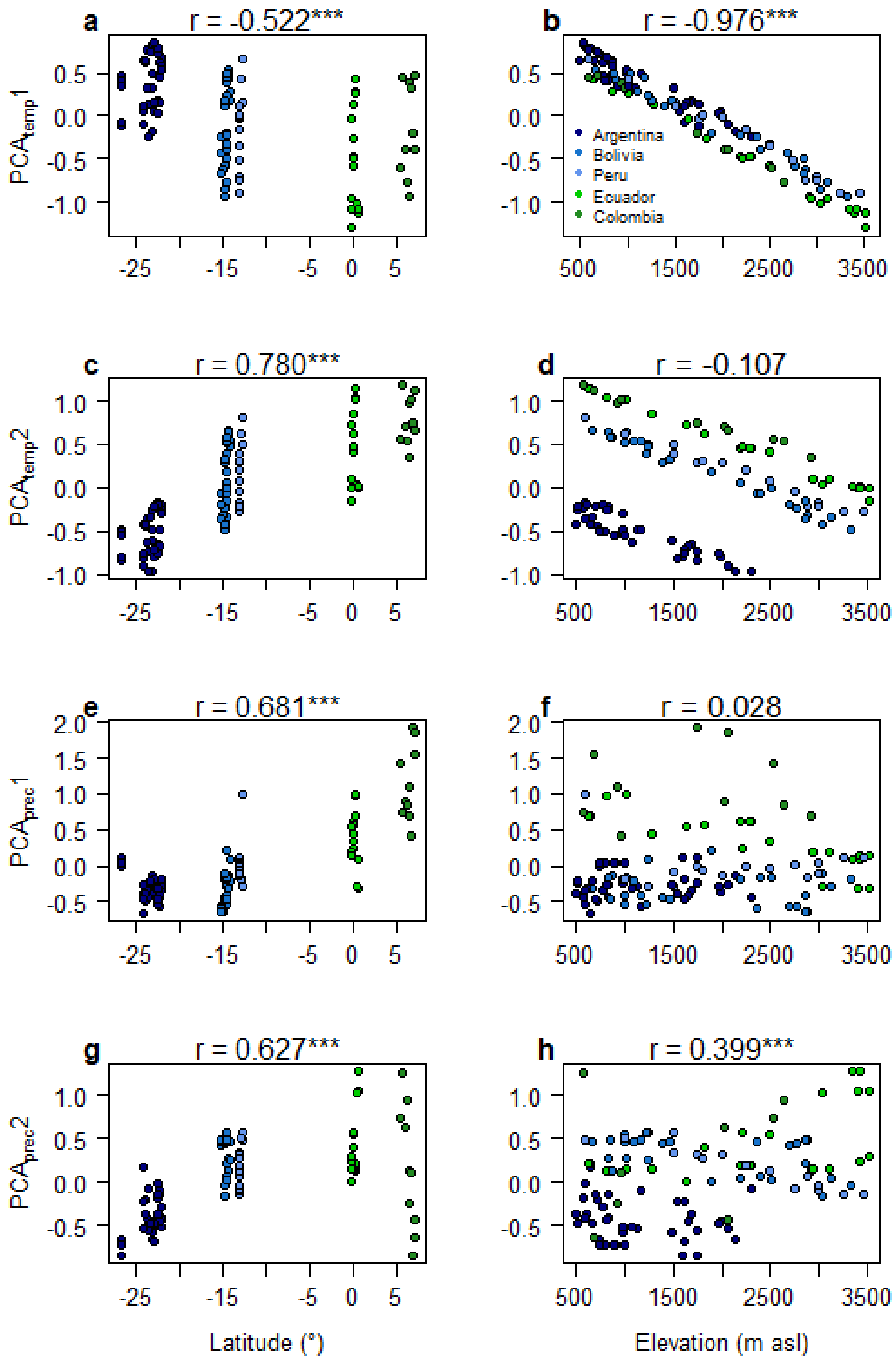

Supplementary Figure 3. Pearson correlation coefficients (r) between the two first axes derived from the principal component analysis (PCA), applied to the data of temperature (11 variables) and the two first PCA axes applied to the precipitation data (8 variables), and the latitudinal (°) and elevational (m asl) variation across the 119 plots established in the subtropical and tropical Andes. The associated probability of the correlations is defined as follows: *: $P \leq 0.05$. **: $P \leq 0.01$. ***: $P \leq 0.001$.

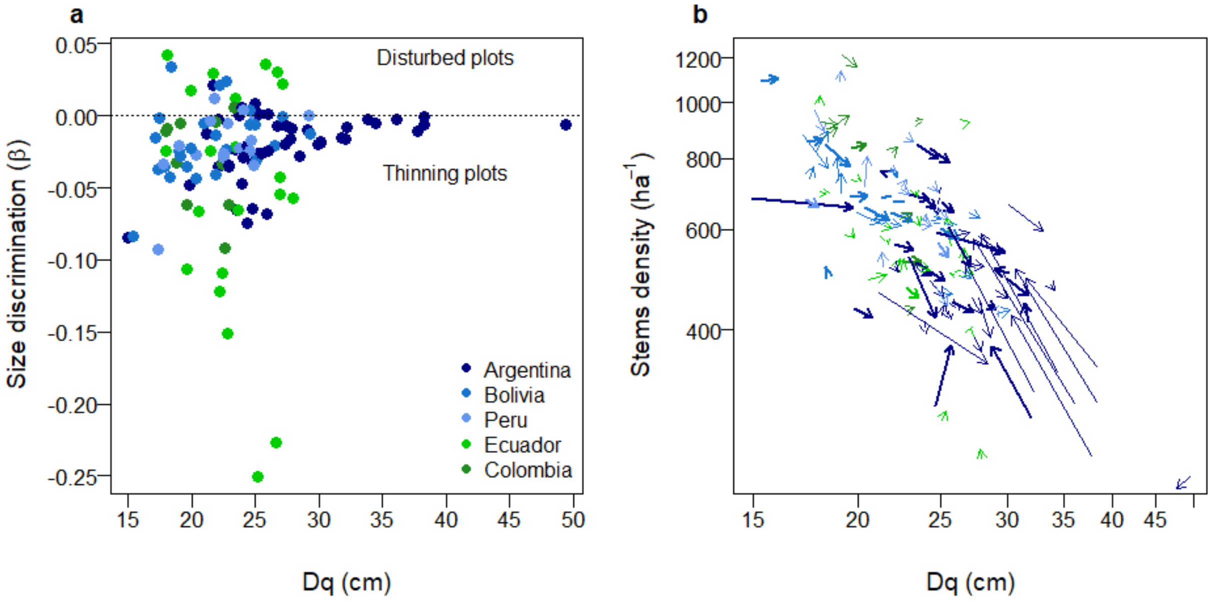

Supplementary Figure 4. a: Size-dependent parameter of mortality (β) derived from the logistic regression (see Main text) employed to differentiate the 119 plots located in the subtropical and tropical Andes along a gradient of disturbance that ranges from sites influenced by competitive thinning (low β) due to post internal disturbance to sites influenced by active disturbance (high β). **b**: Temporal changes in Dq and stem density. Arrows showing an increase in the number of individuals but a decrease in Dq represent disturbed plots where recruitment of juveniles is increasing. Arrows showing an increase in Dq but a decrease in the number of individuals represent competitive thinning.

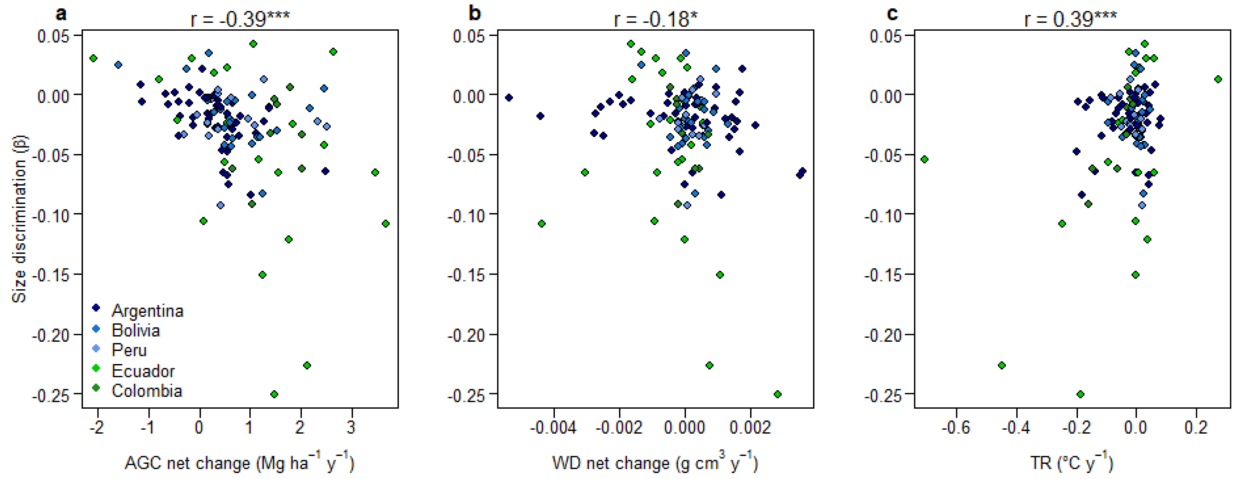

Supplementary Figure 5. Relationship between size-discrimination (β) and AGC net change (**a**), annualized net change in wood density (**b**), and the thermophilization rate (TR) (**c**). r = Pearson correlation coefficient. The associated probability of the correlations is defined as follows: *: $P \leq 0.05$. **: $P \leq 0.01$. ***: $P \leq 0.001$.

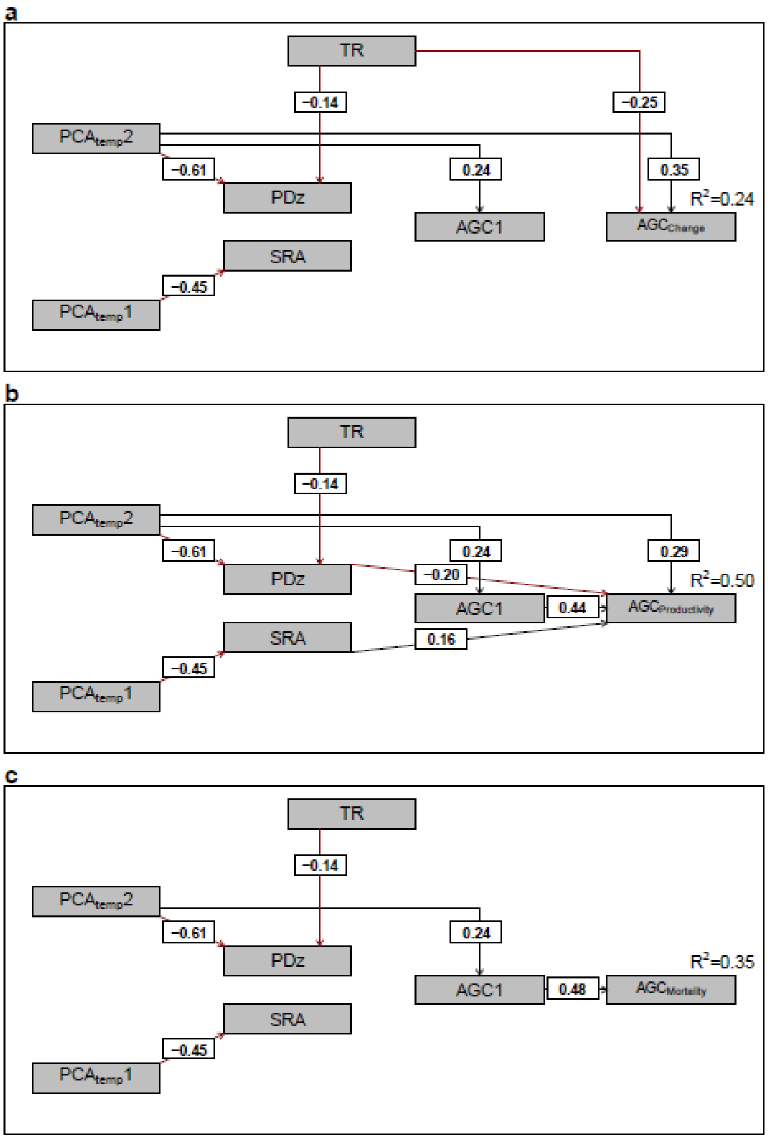

Supplementary Figure 6. Structural equation models (SEM) excluding the size-discrimination parameter (β) that assess the relative importance of climate ($PCA_{temp}1$ and $PCA_{temp}2$), symbiotic root associations (SRA = ln(AM/EcM)), the thermophilization rate (TR; °C y^{-1}), the initial aboveground carbon stock in each plot (AGC1; Mg C ha^{-1}), and the standardize effect size of the phylogenetic diversity (PDz) on determining AGC dynamics (Mg C ha^{-1} y^{-1}). The values over the arrows show the associated linear coefficient between explanatory variables. Black lines represent positive associations, while red lines negative associations. R^2 shows the coefficient of determination of the overall model.

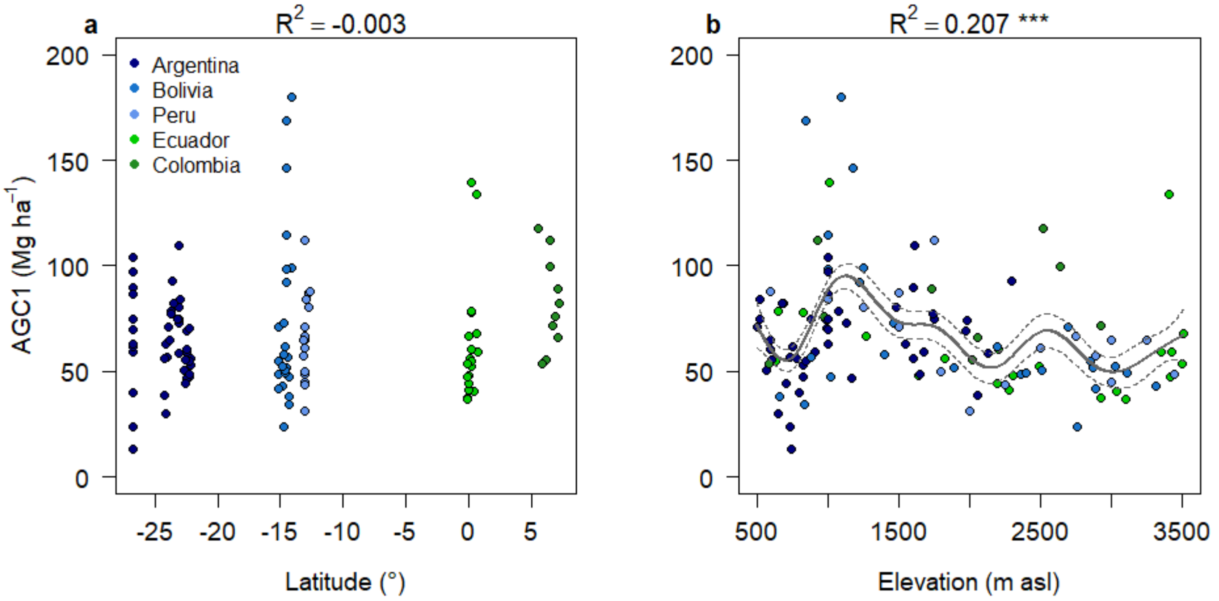

Supplementary Figure 7. Generalized additive models (GAMs) assessing the trend of change of the aboveground (AGC) carbon stocks along latitude (°) (**a**) and elevation (m asl) (**b**), across the 119 plots established in the subtropical and tropical Andes. Negative latitudinal values represent the South hemisphere, while positive latitudinal values represent the North hemisphere. Continuous lines represent the models that were significant. Dashed lines represent the confidence limits (95%). The associated probability of the correlations is defined as follows: *: $P \leq 0.05$. **: $P \leq 0.01$. ***: $P \leq 0.001$. AGC1= carbon stocks in the first census ($Mg\ C\ ha^{-1}$). R^2 = Coefficient of determination of the model.

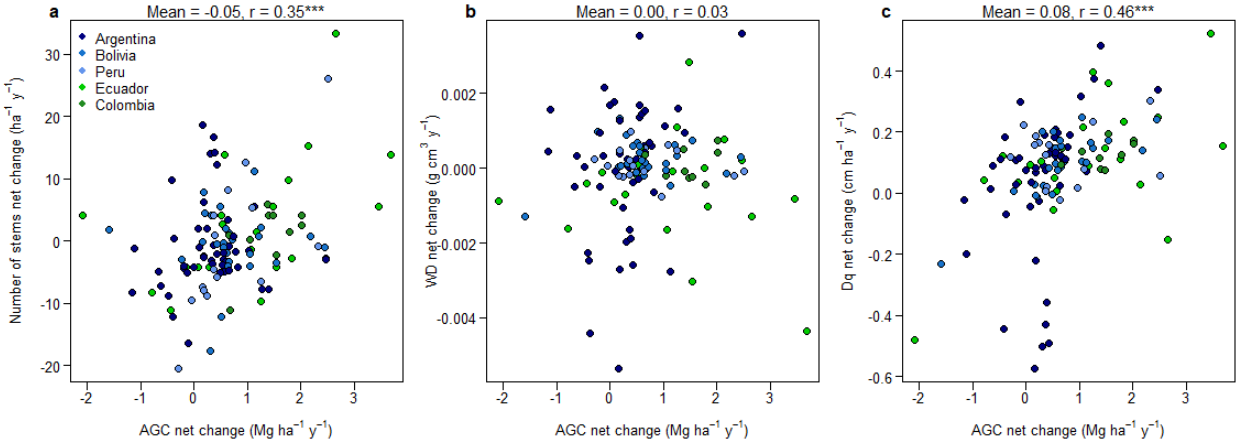

Supplementary Figure 8. Linear correlations between the annualized aboveground carbon (AGC) net change (Mg C ha^{-1} y^{-1}) and the number of stems (**a**), wood density (WD) (**b**), and the quadratic mean diameter (Dq) (**c**) in the subtropical and tropical Andes. Wood density is weighted by the number of individuals. The analyses are made using the 119 plots. r = Pearson correlation coefficient. The associated probability of the correlations is defined as follows: *: $P \leq 0.05$. **: $P \leq 0.01$. ***: $P \leq 0.001$.

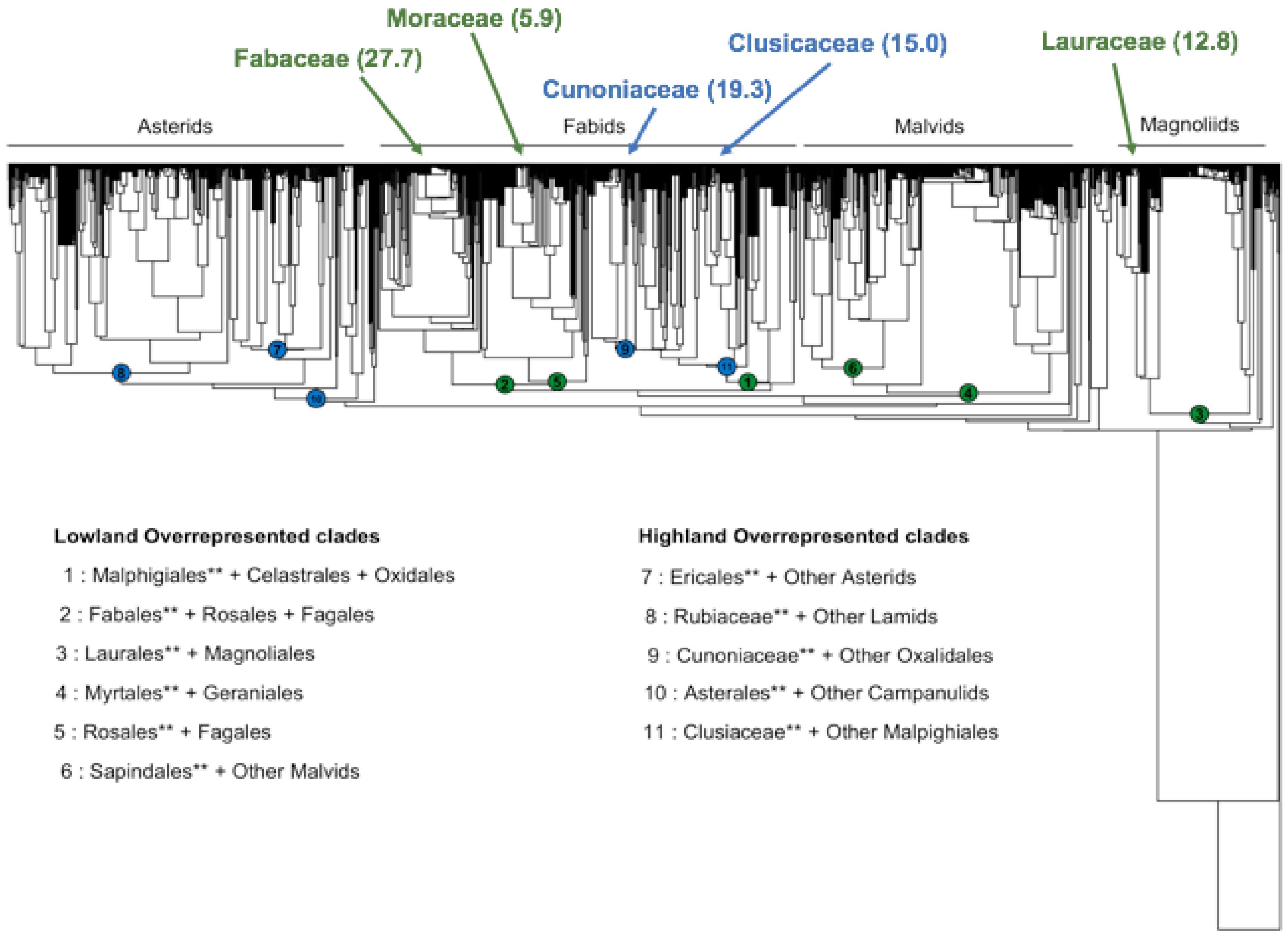

Supplementary Figure 9. Phylogenetic tree of Andean forests using taxa identified at least at the family level. Overrepresented nodes in lowlands (<2000m asl) and highlands (> 2000m asl) are highlighted with green and blue circles, respectively. Node overrepresentation indicates high species richness of one of the two compared sister clades in a particular node. The legend showed taxa identity in each node, and the richest clade was represented by its significance (***). The position in the phylogenetic tree of the families with the highest AGC productivity was pointed in the upper part of the phylogeny. This analysis was run using *Node_analysis* function of the nodiv R package. Most of the clades with the highest AGC productivity were imbedded within any of the overrepresented clades, except the Podocarpaceae family. The latter is an abundant clade only in the subtropical plots, but its importance decreases markedly with latitude.

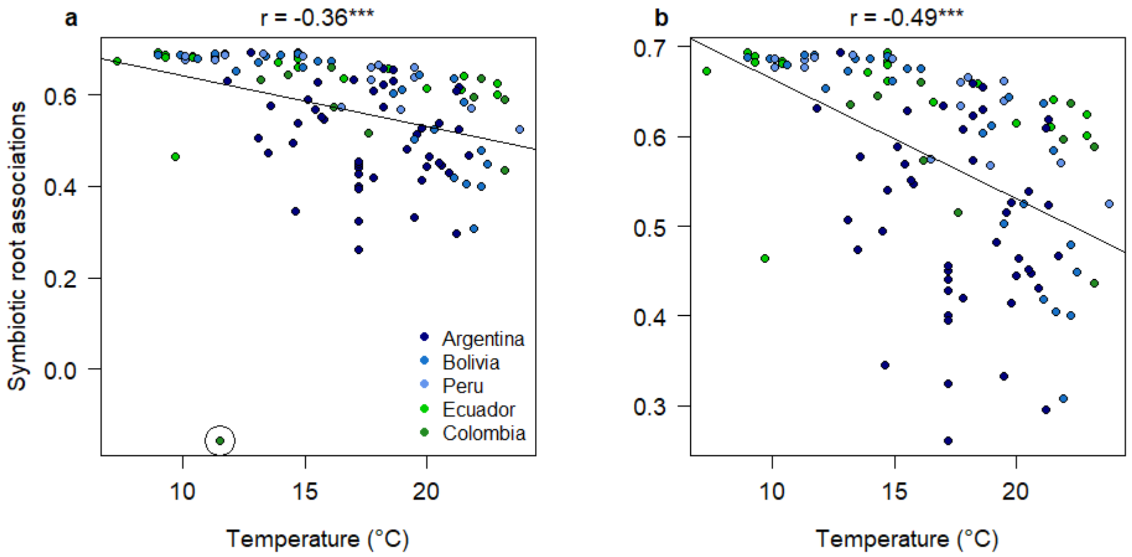

Supplementary Figure 10. Proportional abundances of arbuscular mycorrhizal (AM) and Ectomycorrhizal (EcM) tress across changes in temperature in the subtropical and tropical Andes. **a**: all plots. **b**: excluding the highest plot in Colombia (Belmira; - circled) where there was an overriding dominance of EcM. r = Pearson correlation coefficient. The associated probability of the correlations is defined as follows: *: $P \leq 0.05$. **: $P \leq 0.01$. ***: $P \leq 0.001$.

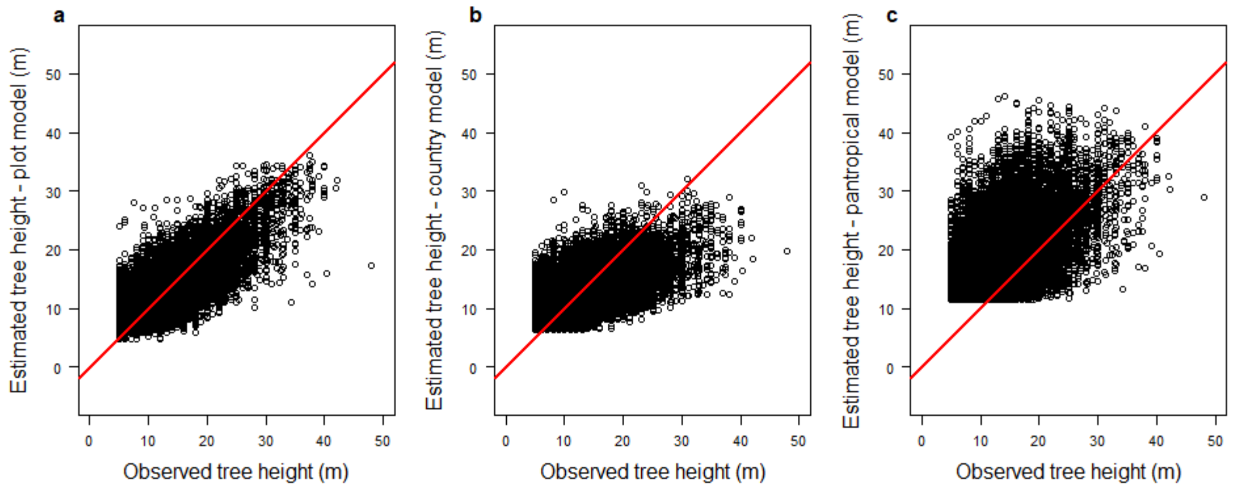

Supplementary Figure 11. Estimated tree heights (based on H:DBH allometric model) and observed tree heights at three different spatial scales: **a**: plot scale; **b**: country; **c**: pantropical model of Feldpausch[47].

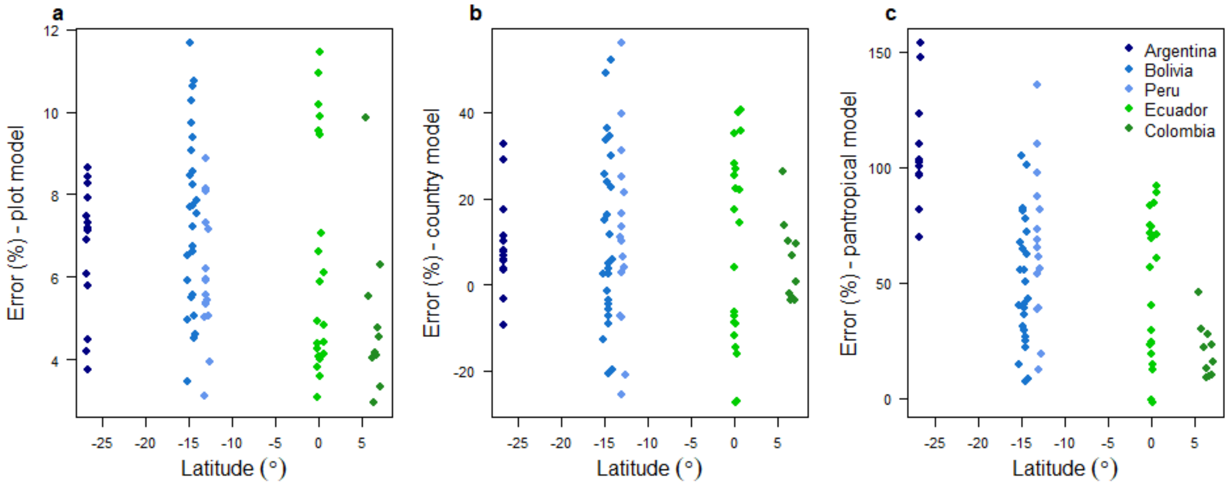

Supplementary Figure 12. Relative errors in the estimation of tree height at the plot (**a**), country (**b**) and pantropical (**c**) scales along the latitudinal gradient. Error (%) was assessed as (100 × (Estimated – Observed) / Observed). Note the difference in scales for the y-axis.

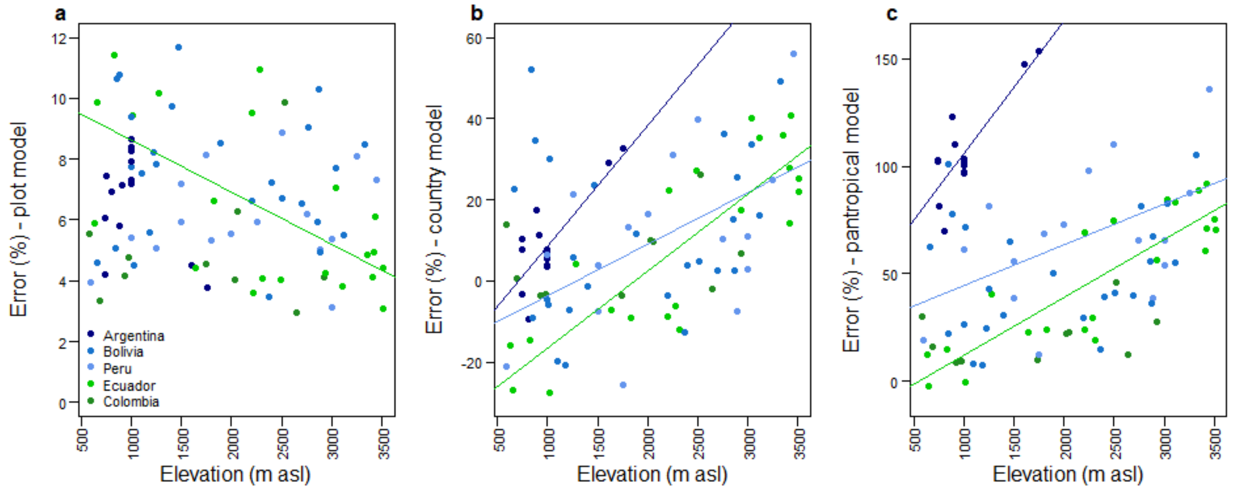

Supplementary Figure 13. Relative errors in the estimation of tree heights at the plot (**a**), country (**b**) and pantropical (**c**) scales along elevational gradients. Error (%) was assessed as (100 × (Estimated – Observed) / Observed). Note the difference in scales for the y-axis. Continuous lines represent the countries in which the relationship between error (%) and elevation was significant ($p \leq 0.05$).

Supplementary Tables

Supplementary Table 1. Synthesis of the definition, hypothesis addressed, and main conclusion of the main explanatory variables of the aboveground carbon (AGC) dynamics (AGC net change, AGC productivity, and AGC mortality) in 119 forest plots located across the subtropical and tropical Andes. See the Methods section for a more detailed description of the variables.

Variable	Code	Definition	Hypothesis	Main conclusion
Elevational climatic variation (°C)	$PCA_{temp}1$	Variation in temperature along elevation	Decreases in temperature along elevation decreases AGC mortality and AGC productivity, and thus AGC net change	$PCA_{temp}1$ did not significantly determined AGC net change, AGC productivity, or AGC mortality at a regional scale
Latitudinal climatic variation (°C)	$PCA_{temp}2$	Variation in temperature along latitude	The increase in mean annual temperature as well as decrease in daily variation and seasonality of MAT and PA towards the equator increases AGC net change	$PCA_{temp}2$ did significantly determined AGC net change and AGC productivity, but AGC mortality at regional scale
Initial aboveground carbon stock (Mg C ha^{-1})	AGC1	Aboveground carbon stored in the living aboveground biomass in the first census	AGC dynamics increases along with the increase of the initial AGC stocks	The larger the initial amount of AGC the larger the AGC net change, AGC productivity, and AGC mortality
Thermophilization rate (°C)	TR	Directional changes in species composition due to changes in the plot mean thermal optimum	Higher mortality of species inhabiting the lower	The indirect negative effect of TR on C stocks likely reflects the loss of individuals in the hotter portion of their species' thermal ranges

Variable	Code	Definition	Hypothesis	Main conclusion
Symbiotic root associations	SRA	Log (AM/EcM) in each plot Arbuscular mycorrhizal (AM) trees, Ectomycorrhizal (EcM) trees	Enhanced nutrient absorption by AM fungi increases AGC productivity and lowers tree mortality, thereby enhancing AGC net change	SRA positively affect AGC productivity by improving nutrient cycling in AM-dominated forests, which drives more rapid plant growth
Phylogenetic diversity	PDz	Standardized effect size of the phylogenetic diversity in each plot	Niche complementarity in resource use by functionally-different clades increase productivity of species assemblages	Selection effects and the conservation of large stature within just a few key clades with different evolutionary histories play an important role in driving AGC productivity in Andean forests
Size-dependent probability of mortality	β	β parameter assessed from the probabillty of death (P) as a function of DBH using logistic regression	The more disturbed a plot is the more AGC it will gain	Recovering from disturbance plays a key role in determining AGC gains, while the death of large trees drives AGC losses

Supplementary Table 2. Mean (± standard error) coarse woody aboveground biomass carbon (AGC; DBH ≥ 10 cm) stocks and dynamics (Mg C ha^{-1} y^{-1}) across countries in the subtropical and tropical Andes. Significant differences between countries are denoted by: *: P ≤ 0.05. **: P ≤ 0.01. ***: P ≤ 0.001. ns: non-significant. In case of significant differences, a Tukey Honest Significant test was employed to differentiate the means. Mean values with different letters indicate significant differences. AGC net change = aboveground carbon net change (Mg C ha^{-1} y^{-1}). AGC recruitment = aboveground carbon recruitment (Mg ha^{-1} y^{-1}). AGC growth = aboveground carbon growth (Mg C ha^{-1} y^{-1}). AGC productivity = aboveground carbon productivity (Mg C ha^{-1} y^{-1}). AGC mortality = aboveground carbon mortality (Mg C ha^{-1} y^{-1}). AGC1 = mean initial carbon stock (Mg C ha^{-1}). AGC_{final} = mean final carbon stock (Mg C ha^{-1}).

	**Argentina**	**Bolivia**	**Colombia**	**Ecuador**	**Peru**	**Total**
**Number of plots**	46[+]	26	10	21	16	119
**Plot area rank (ha)**	0.32 – 1.28	1	1	0.36	1	
**AGC1**	65.02 ± 2.92	71.47 ± 7.99	82.44 ± 7.01	62.09 ± 5.99	65.94 ± 5.18	67.50 ± 2.51 ns
**AGC_{final}**	68.62 ± 2.75	76.11 ± 8.49	90.01 ± 6.94	64.29 ± 6.22	68.16 ± 5.23	71.23 ± 2.61 ns
**AGC net change**	0.30 ± 0.10 a	0.67 ± 0.15 ab	1.40 ± 0.15 b	1.10 ± 0.31 b	0.70 ± 0.20 ab	0.67 ± 0.08 **
**AGC recruitment**	0.10 ± 0.01	0.15 ± 0.03	0.09 ± 0.01	0.09 ± 0.03	0.06 ± 0.02	0.10 ± 0.01 ns
**AGC growth**	1.05 ± 0.06 a	1.55 ± 0.18 ab	2.00 ± 0.20 b	1.81 ± 0.22 b	1.60 ± 0.22 ab	1.45 ± 0.08 ***
**AGC productivity**	1.15 ± 0.07 a	1.71 ± 0.19 b	2.09 ± 0.21 b	1.90 ± 0.23 b	1.66 ± 0.23 ab	1.55 ± 0.08 ***
**AGC mortality**	0.94 ± 0.06	1.19 ± 0.16	0.89 ± 0.15	0.98 ± 0.18	1.10 ± 0.17	1.02 ± 0.06 ns

[+]**:** Thirty-nine (39) plots in Argentina have 1-ha.

Supplementary Table 3. Information theoretic (IT) modeling employing model-based inference to generate a set of candidate models that represent competing hypotheses build up by different sets of explanatory variables that explain aboveground carbon (AGC) dynamics. The competing hypotheses were represented by climate (PCA_{temp1} and PCA_{temp2}), symbiotic root associations (SRA = ln(AM/EcM)), the thermophilization rate (TR; °C y^{-1}), the initial aboveground carbon stock in each plot (AGC1; Mg C ha^{-1}), the standardize effect size of the phylogenetic diversity (PDz), and the size-dependent parameter (β) of mortality. MAE prom: model-averaged coefficient estimate. MAE SE: unconditional standard error. P: probability. RVI: relative variable importance. No. Model: Number of models that include the variable. Values in bold shows significant variables.

	**Variable/ Parameter**	**MAE prom**	**MAE SE**	**P**	**RVI**	**No. Model**
**AGC net change (Mg C ha^{-1} y^{-1})**	Intercept	0.000	0.000			
	AGC1	0.153	0.080	0.059	0.72	13
	β	**-0.309**	**0.090**	**0.001**	**1.00**	21
	$PCA_{temp}1$	-0.053	0.081	0.518	0.23	7
	$PCA_{temp}2$	**0.292**	**0.099**	**0.003**	**1.00**	21
	TR	-0.121	0.080	0.136	0.51	10
	PDz	-0.073	0.080	0.367	0.30	8
	SRA	0.049	0.081	0.547	0.23	7
**AGC productivity (Mg C ha^{-1} y^{-1})**	Intercept	0.000	0.000			
	AGC1	**0.436**	**0.068**	**0.000**	**1.00**	9
	β	-0.042	0.068	0.539	0.22	3
	$PCA_{temp}1$	0.018	0.073	0.811	0.21	3
	$PCA_{temp}2$	**0.239**	**0.081**	**0.004**	**1.00**	9
	TR	-0.021	0.068	0.757	0.11	1
	PDz	**-0.155**	**0.068**	**0.024**	**0.94**	8
	SRA	**0.136**	**0.068**	**0.047**	**0.79**	6
**AGC mortality (Mg C ha^{-1} y^{-1})**	Intercept	0.000	0.000			
	AGC1	**0.350**	**0.071**	**0.000**	**1.00**	14
	β	**0.387**	**0.079**	**0.000**	**1.00**	14
	$PCA_{temp}1$	0.070	0.075	0.357	0.28	5
	$PCA_{temp}2$	0.093	0.087	0.287	0.36	7
	TR	**0.144**	**0.071**	**0.044**	**0.81**	10
	PDz	**-0.166**	**0.077**	**0.033**	**0.84**	11
	SRA	0.123	0.072	0.090	0.65	9

Supplementary Table 4. Loadings associated to the first two axes of the principal component analysis (PCA) applied to temperature and precipitation data according to CHELSA[46]. The abbreviations of the climatic variables are described in the Materials and Methods section. In bold are the two most extreme values along each axis.

	**Variable**	**PCA 1**	**PCA 2**
**Temperature**	MAT	1.6472	0.7449
	MDR	0.8397	-1.4986
	Isoth	**-1.0234**	1.4260
	TS	1.0569	-1.4504
	MaxTWarmM	**1.8065**	-0.0404
	MinTCM	0.6589	**1.6842**
	TAR	0.9909	**-1.5118**
	MeanTWetQ	1.8034	0.1298
	MeanTDQ	1.1378	1.3973
	MeanTWwarmQ	1.8037	0.1081
	MeanTCQ	0.9601	1.5273
**Precipitation**	AP	**1.9151**	-0.2468
	PWetM	1.6480	-1.0432
	PDM	1.7647	**0.7752**
	PS	**-1.0777**	**-1.5392**
	PWetQ	1.7037	-0.9510
	PDQ	1.7908	0.7519
	PWarmQ	1.4398	-1.2776
	PCQ	1.5962	0.8010

Supplementary Table 5. Information theoretic (IT) modeling employing model-based inference to generate a set of candidate models that represent competing hypotheses of different sets of explanatory variables (excluding β) explaining aboveground carbon (AGC) dynamics. The competing hypotheses were represented by climate (PCA_{temp1} and PCA_{temp2}), symbiotic root associations (SRA = ln(AM/EcM)), the thermophilization rate (TR; °C y^{-1}), the initial aboveground carbon stock in each plot (AGC 1; C Mg ha^{-1}), and the standardized effect size of the phylogenetic diversity (PDz). nAGC = aboveground carbon net change (Mg C ha^{-1} y^{-1}). pAGC = aboveground carbon productivity (Mg C ha^{-1} y^{-1}). mAGC = aboveground carbon mortality (Mg C ha^{-1} y^{-1}). MAE prom: model-averaged coefficient estimate. MAE SE: unconditional standard error. P: probability. RVI: relative variable importance. No. Model: Number of models that include the variable. Values in bold shows significant variables.

	Variable/ Parameter	MAE prom	MAE SE	P	RVI	No. Model
**AGC net change (Mg C ha^{-1} y^{-1})**	Intercept	0.000	0.000			
	AGC 1	0.077	0.083	0.353	0.32	4
	PCAtemp 1	-0.034	0.083	0.685	0.20	3
	PCAtemp 2	**0.378**	**0.096**	**0.000**	**1.00**	10
	TR	**-0.253**	**0.082**	**0.002**	**1.00**	10
	PDz	-0.046	0.083	0.584	0.25	4
	SRA	0.041	0.083	0.624	0.21	3
**AGC productivity (Mg C ha-1 y-1)**	Intercept	0.000	0.000			
	AGC 1	**0.436**	**0.068**	**0.000**	**1.00**	6
	PCAtemp 1	0.013	0.074	0.861	0.21	2
	PCAtemp 2	**0.246**	**0.083**	**0.003**	**1.00**	6
	TR	-0.021	0.068	0.757	0.14	1
	PDz	**-0.154**	**0.068**	**0.025**	**0.92**	5
	SRA	**0.135**	**0.068**	**0.049**	**0.79**	4
**AGC mortality (Mg C ha-1 y-1)**	Intercept	0.000	0.000			
	AGC 1	**0.439**	**0.078**	**0.000**	**1.00**	12
	PCAtemp 1	0.055	0.082	0.512	0.25	4
	PCAtemp 2	-0.016	0.092	0.860	0.26	5
	TR	**0.323**	**0.079**	**0.000**	**1.00**	12
	PDz	**-0.159**	**0.077**	**0.042**	**0.77**	7
	SRA	0.122	0.078	0.121	0.56	7

Supplementary Table 6. List of the families per elevational band that contribute to ~50% of the total AGC productivity (Mg C ha^{-1} y^{-1}) in the subtropical and tropical Andes. The contribution was estimated for all plots and separating subtropical and tropical plots. The accumulative percentage of annual productivity per family was included. Node number indicates the overrepresented node (and clade identity) in which the family is embedded (see Supplementary Figure 8).

Region	500-1200			1200-2000			2000-2800			2800-3600		
	Family	Prod (%)	Node	Family	Prod (%)	Node	Family	Prod (%)	Node	Family	Prod (%)	Node
All	Fabaceae	27.7	2	Myrtaceae	10.5	4	Lauraceae	15.4		Cunoniaceae	19.3	11
	Lauraceae	40.5	3	Podocarpaceae	19.3		Podocarpaceae	24.1		Melastomataceae	36.5	
	Moraceae	46.4	5	Fabaceae	27.4	2	Euphorbiaceae	32.3		Clusiaceae	51.5	9
	Myrtaceae	52.3	4	Lauraceae	34.7	3	Clusiaceae	41.0	11			
				Aquifoliaceae	40.1		Cunoniaceae	47.3	9			
				Meliaceae	45.0	6	Melastomataceae	53.2				
				Elaeocarpaceae	49.0							
				Moraceae	53.0	5						
Sub-tropical	Fabaceae	25.6	2	Myrtaceae	18.8	4	Podocarpaceae	53.6				
	Lauraceae	44.3	3	Podocarpaceae	35.0							
	Myrtaceae	52.8	4	Aquifoliaceae	45.0							
				Meliaceae	54.1	6						
Tropical	Fabaceae	31.5	5	Lauraceae	13.1	3	Lauraceae	17.6		Cunoniaceae	19.3	11
	Moraceae	39.2	5	Fabaceae	21.0	2	Euphorbiaceae	27.0		Melastomataceae	36.5	
	Malvaceae	45.8		Moraceae	28.5	5	Clusiaceae	35.8	11	Clusiaceae	51.5	9
	Petiveriaceae	51.0		Sapotaceae	35.1		Cunoniaceae	44.1	9			
				Rubiaceae	41.4	8	Melastomataceae	50.8				
				Melastomataceae	46.7							
				Anacardiaceae	51.7	6						

Supplementary Table 7. Height-diameter (H:DBH) allometric models evaluated using the modelHD function available in the BIOMASS R Package[44]. a, b, c, and d are the model parameters.

Model	
Log1	$\ln(H) = a + b \times \ln(DBH)$
Log2	$\ln(H) = a + b \times \ln(DBH) + c \times \ln(DBH)^2$
Weibull	$H = a \times (1-\exp(-(DBH/b)^c))$
Michaelis-Menten	$H = (a \times DBH) / (b + DBH)$

Supplementary Table 8. Plot-based H:DBH allometries fitted for the 119 permanent plots surveyed in the subtropical and tropical Andes. The best model was the one that minimizes the Residual Standard Error (RSE). Model selection was performed using the BIOMASS library for R[44] (see Methods). The structure of the four models employed is presented in Supplementary Table 7.

Plot	Parameters			Selected model	RSE	RSElog
	a	b	c			
ab	22.316	44.978	0.723	weibull_arg	2.787	0.000
ai	22.316	44.978	0.723	weibull_arg	2.787	0.000
Angelopolis	30.341	41.507	0.583	weibull	2.536	2.536
Anori	1.435	0.455	0.000	log1	3.320	0.211
ap	22.316	44.978	0.723	weibull_arg	2.787	0.000
ba	22.316	44.978	0.723	weibull_arg	2.787	0.000
ba_a	22.316	44.978	0.723	weibull_arg	2.787	0.000
Belmira	20.063	13.558	1.081	weibull	3.254	3.254
bla	22.316	44.978	0.723	weibull_arg	2.787	0.000
bmI	22.316	44.978	0.723	weibull_arg	2.787	0.000
bmII	22.316	44.978	0.723	weibull_arg	2.787	0.000
Bosque de mirtaceas	0.712	0.423	0.000	log1	1.742	0.194
Bosque de mirtaceas, ladera sur	31.338	181.798	0.576	weibull	1.995	1.995
Caicedo	1.648	0.374	0.000	log1	2.570	0.170
CAL-01	21.752	21.315	1.211	weibull	2.885	2.885
CAL-02	22.134	26.907	1.230	weibull	2.348	2.348
cb	22.316	44.978	0.723	weibull_arg	2.787	0.000
cc	22.316	44.978	0.723	weibull_arg	2.787	0.000
Cebil	16.523	16.234	1.207	weibull	2.769	2.769
ECCA_GUAN_01	16.519	8.670	0.000	michaelis	2.115	2.115
ECCA_GUAN_02	18.268	9.712	0.000	michaelis	2.312	2.312
ECCA_PAGR_01	-0.153	1.318	-0.171	log2	2.316	0.261
ECCA_VINE_01	16.925	12.737	0.000	michaelis	2.029	2.029
ECCA_VINE_02	20.439	20.444	0.000	michaelis	2.499	2.499
ECPI_BECL_01	18.964	12.193	1.367	weibull	2.649	2.649
ECPI_BECL_02	22.164	15.577	1.111	weibull	4.148	4.148
ECPI_BECL_03	40.379	29.921	0.000	michaelis	4.312	4.312
ECPI_CEDR_01	12.332	9.998	1.305	weibull	1.966	1.966
ECPI_CEDR_03	19.758	14.928	0.000	michaelis	2.061	2.061
ECPI_INTI_02	23.461	17.671	1.182	weibull	3.823	3.823
ECPI_MALO_01	25.600	16.509	1.262	weibull	4.809	4.809
ECPI_MALO_02	35.975	23.654	1.007	weibull	4.841	4.841

Plot	Parameters			Selected model	RSE	RSElog
	a	b	c			
ECPI_MAPI_01	49.572	28.423	0.000	michaelis	5.327	5.327
ECPI_MAPI_02	39.966	26.487	0.000	michaelis	3.315	3.315
ECPI_MIND_01	31.462	23.989	0.000	michaelis	3.915	3.915
ECPI_RIBR_01	16.115	10.468	1.922	weibull	2.581	2.581
ECPI_VERD_01	18.549	14.037	0.000	michaelis	2.214	2.214
ECPI_VERD_02	13.198	10.603	1.525	weibull	2.076	2.076
ECPI_VERD_03	14.159	8.080	0.000	michaelis	1.726	1.726
ECPI_YANA_01	0.119	1.225	-0.155	log2	1.747	0.173
es	22.316	44.978	0.723	weibull_arg	2.787	0.000
ESP-01	-0.805	1.857	-0.241	log2	2.553	0.219
Guaran	3.574	-1.547	0.352	log2	1.550	0.204
Jardin	26.769	18.941	0.000	michaelis	4.148	4.148
km25	22.316	44.978	0.723	weibull_arg	2.787	0.000
km34	22.316	44.978	0.723	weibull_arg	2.787	0.000
Ladera Norte	36.614	120.832	0.694	weibull	2.681	2.681
Ladera Sur	53.028	523.326	0.539	weibull	2.483	2.483
lc	22.316	44.978	0.723	weibull_arg	2.787	0.000
li	22.316	44.978	0.723	weibull_arg	2.787	0.000
ma	22.316	44.978	0.723	weibull_arg	2.787	0.000
Maceo	1.537	0.418	0.000	log1	3.005	0.201
me	22.316	44.978	0.723	weibull_arg	2.787	0.000
mo	22.316	44.978	0.723	weibull_arg	2.787	0.000
Mora	2.436	-0.599	0.167	log2	2.158	0.244
ms	22.316	44.978	0.723	weibull_arg	2.787	0.000
no	22.316	44.978	0.723	weibull_arg	2.787	0.000
Nogal+Cedro	0.528	0.598	0.000	log1	2.774	0.267
PAN-02	28.071	25.788	0.732	weibull	2.999	2.999
ph	22.316	44.978	0.723	weibull_arg	2.787	0.000
Porce	1.300	0.503	0.000	log1	3.222	0.213
PP_Chaqui_31	22.989	12.656	0.000	michaelis	3.850	3.850
PP_Chaqui_32	16.020	14.112	1.113	weibull	2.535	2.535
PP_Chiriu_2	21.605	13.478	0.000	michaelis	3.398	3.398
PP_Jucuma_35	1.192	0.363	0.000	log1	2.786	0.294
PP_Kañupa_44	12.663	12.754	0.999	weibull	2.832	2.832
PP_Pintat_5	784.994	25267.954	0.596	weibull	3.275	3.275
PP_Resina_12	2.247	-0.181	0.067	log2	2.029	0.211
PP_Resina_13	0.840	0.442	0.000	log1	1.834	0.223
PP_Resina_14	15.135	9.396	0.000	michaelis	1.893	1.893

Plot	Parameters			Selected model	RSE	RSElog
	a	b	c			
PP_Sanmar_21	32.340	23.775	0.966	weibull	4.361	4.361
PP_Sanmar_22	32.784	27.325	0.000	michaelis	3.421	3.421
PP_Sumpul_33	29.402	26.423	0.951	weibull	3.909	3.909
PP_Sumpul_34	-0.712	1.727	-0.192	log2	4.320	0.281
PP_Tapuri_45	-0.722	1.903	-0.260	log2	2.998	0.246
PP_Tapuri_46	-0.197	1.668	-0.234	log2	2.225	0.182
PP_Terraz_41	18.177	22.571	0.690	weibull	2.189	2.189
PP_Tintay_24	20.810	15.470	0.839	weibull	3.648	3.648
PP_Tintay_25	23.454	32.170	0.700	weibull	3.613	3.613
PP_Titiri_42	19.378	11.704	0.000	michaelis	2.528	2.528
PP_Tocoaq_28	30.298	20.731	0.000	michaelis	3.168	3.168
PP_Tocoaq_29	19.047	15.051	1.010	weibull	3.174	3.174
PP_Tocoaq_30	22.332	13.058	0.000	michaelis	3.042	3.042
PP_Waturu_43	18.315	24.728	0.619	weibull	2.907	2.907
PP_Yarimi_10	42.349	33.391	0.000	michaelis	4.145	4.145
PP_Yarimi_11	29.370	20.151	1.125	weibull	4.057	4.057
PP_Yarimi_9	36.074	31.477	1.017	weibull	4.690	4.690
rsI	22.316	44.978	0.723	weibull_arg	2.787	0.000
rsII	22.316	44.978	0.723	weibull_arg	2.787	0.000
sa	22.316	44.978	0.723	weibull_arg	2.787	0.000
Segovia	54.642	132.365	0.563	weibull	2.968	2.968
sm	22.316	44.978	0.723	weibull_arg	2.787	0.000
SPD-01	28.844	12.929	0.000	michaelis	3.984	3.984
SPD-02	26.930	18.965	0.000	michaelis	3.078	3.078
Superplot-ha1	18.701	33.042	0.659	weibull	2.700	2.700
Superplot-ha2	0.850	0.461	0.000	log1	3.310	0.282
Superplot-ha3	847.161	192258.285	0.488	weibull	3.075	3.075
Superplot-ha4	19.481	30.211	0.847	weibull	2.982	2.982
Superplot-ha5	17.259	21.211	1.033	weibull	2.926	2.926
Superplot-ha6	26.915	34.291	0.000	michaelis	3.503	3.503
ta	22.316	44.978	0.723	weibull_arg	2.787	0.000
Tamesis	1.027	0.539	0.000	log1	3.724	0.234
te	22.316	44.978	0.723	weibull_arg	2.787	0.000
to	22.316	44.978	0.723	weibull_arg	2.787	0.000
TON-02	23.286	25.125	1.228	weibull	2.540	2.540
TRU-01	18.023	38.103	0.748	weibull	2.422	2.422
TRU-02	18.660	23.562	1.046	weibull	2.862	2.862

Plot	Parameters			Selected model	RSE	RSElog
	a	b	c			
TRU-03	21.378	14.717	0.000	michaelis	2.689	2.689
TRU-04	17.735	18.060	1.114	weibull	2.702	2.702
TRU-05	18.347	24.551	1.340	weibull	3.205	3.205
TRU-06	18.002	24.265	1.086	weibull	2.461	2.461
TRU-07	23.158	22.428	0.000	michaelis	2.285	2.285
TRU-08	31.783	62.277	0.732	weibull	2.278	2.278
Ventanas	1.201	0.496	0.000	log1	3.679	0.248
vm	22.316	44.978	0.723	weibull_arg	2.787	0.000
WAY-01	19.470	25.170	0.760	weibull	1.697	1.697
yu	22.316	44.978	0.723	weibull_arg	2.787	0.000
za	22.316	44.978	0.723	weibull_arg	2.787	0.000
za_a	22.316	44.978	0.723	weibull_arg	2.787	0.000

Supplementary Table 9. Results of a Structural Equation Modeling (SEM) applied to only the abiotic explanatory variables of AGC dynamics in Andean forests. The climatic variables employed were the four principal component axes derived from temperature (PCA_{temp}) and precipitation (PCA_{prec}) (see main text). The results show the lack of significance of any of the abiotic variables employed due to collinearity between climatic variables (see Supplementary Figure 2).

Response	Variable	Coefficient	Std. Error	P-value	R^2
**AGC net change**	**AGC1**	0.108	0.085	0.201	0.217
	**$PCA_{temp}1$**	-0.045	0.128	0.724	
	**$PCA_{temp}2$**	0.256	0.191	0.179	
	**$PCA_{prec}1$**	0.226	0.148	0.127	
	**$PCA_{prec}2$**	0.000	0.176	0.999	
**AGC1**	**$PCA_{temp}1$**	**0.296**	**0.131**	**0.024**	0.090
	**$PCA_{temp}2$**	-0.008	0.207	0.969	
	**$PCA_{prec}1$**	0.182	0.159	0.253	
	**$PCA_{prec}2$**	0.213	0.187	0.254	
**AGC productivity**	**AGC1**	**0.421**	**0.066**	**0.000**	0.468
	**$PCA_{temp}1$**	0.100	0.105	0.339	
	**$PCA_{temp}2$**	0.226	0.158	0.152	
	**$PCA_{prec}1$**	0.163	0.123	0.185	
	**$PCA_{prec}2$**	0.205	0.144	0.154	
**AGC1**	**$PCA_{temp}1$**	**0.296**	**0.131**	**0.024**	0.090
	**$PCA_{temp}2$**	-0.008	0.207	0.969	
	**$PCA_{prec}1$**	0.182	0.159	0.253	
	**$PCA_{prec}2$**	0.213	0.187	0.254	
**AGC mortality**	**AGC1**	**0.387**	**0.078**	**0.000**	0.258
	**$PCA_{temp}1$**	0.177	0.123	0.151	
	**$PCA_{temp}2$**	-0.006	0.187	0.976	
	**$PCA_{prec}1$**	-0.103	0.145	0.479	
	**$PCA_{prec}2$**	0.273	0.169	0.106	
**AGC1**	**$PCA_{temp}1$**	**0.296**	**0.131**	**0.024**	0.090
	**$PCA_{temp}2$**	-0.008	0.207	0.969	
	**$PCA_{prec}1$**	0.182	0.159	0.253	
	**$PCA_{prec}2$**	0.213	0.187	0.254	
